# Approaches to assessing completeness of colorectal polyp resections in clinical practice: a systematic scoping review

**DOI:** 10.1055/a-2783-3897

**Published:** 2026-02-13

**Authors:** Querijn N. E. van Bokhorst, Silpa Yarra, Manon van der Vlugt, Heiko Pohl, Evelien Dekker, Aasma Shaukat

**Affiliations:** 1 Department of Gastroenterology and Hepatology 522567 Amsterdam University Medical CenterAmsterdamThe Netherlands; 2 571165 Amsterdam Gastroenterology Endocrinology MetabolismAmsterdamThe Netherlands; 3 Cancer Center AmsterdamAmsterdamThe Netherlands; 4 Division of Gastroenterology 12296 NYU Grossman School of MedicineNew YorkNew YorkUnited States; 5 Department of Gastroenterology and Hepatology 20127 White River Junction VA Medical CenterWhite River JunctionVermontUnited States

## Abstract

**Background:**

Protocols for standardized assessment of complete colorectal polyp resection are lacking, contributing to divergent quality standards and hindering reliable comparison of incomplete resection rates (IRRs) across resection devices, techniques, endoscopists, and institutions. We reviewed available methods to inform the development of such protocols.

**Methods:**

We systematically searched MEDLINE, Embase, Web of Science, and Cochrane Library databases from inception to 30 July 2024. Studies describing the use or validation of methods for assessing completeness of polyp resection were included. Studies using recurrence detected at follow-up or histopathological resection specimen margin assessment as outcome measures were excluded, unless used as reference standards for evaluation of other methods.

**Results:**

45 eligible studies were identified. Methods for assisting in visual confirmation of complete resection included image enhancement techniques (6 studies), artificial intelligence (1 study), and resection defect diameter (1 study). Methods for measuring IRRs based on a histopathological reference standard involved biopsy sampling (29 studies) and extended margin resection (8 studies). IRR measurement protocols differed in terms of factors such as location and number of biopsies (1–8) and widths of extended resections (1–3 mm). IRRs >10% were observed for all polyp size categories and almost all resection techniques, with considerable variability in IRRs reported across studies (biopsy sampling 0–24.2%; extended resection 0–61.1%).

**Conclusions:**

Different methods are available to assist in visual confirmation of complete resection and measuring IRRs, with considerable variability in their application, highlighting the need for standardized assessment of complete colorectal polyp resection.

## Abstract (de)

**Graphical Abstract:**

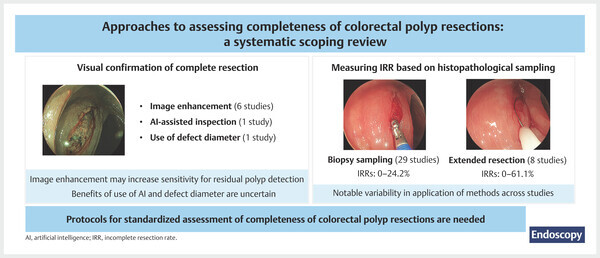

## Introduction

Incomplete resection of neoplastic colorectal polyps, defined as resections that leave (microscopic) residual polyp tissue at the border (lateral margin) or the base (basal margin) of a polyp resection defect 1 , can lead to polyp recurrence and post-colonoscopy colorectal cancer (PCCRC). Incomplete polyp resections are estimated to be responsible for 7%–19% of post-colonoscopy colorectal cancers 2 3 4 5 6 . Moreover, recurrent polyps are often more difficult to treat due to fibrosis and tissue tethering at the resection site. This increases the risk of repeated incomplete resections and resection-related adverse events 7 .

Despite the clinical relevance of complete polyp resections, standardized approaches for evaluation of the completeness of polyp resection are lacking. Consequently, standard practices for visual confirmation of a presumed complete resection are likely to vary among individual endoscopists and across institutions. This may contribute to divergent quality standards for colorectal polypectomies. In addition, the lack of clinical standards results in the use of divergent approaches for measuring the incomplete polyp resection rate (IRR) 8 , defined as the proportion of polyps in which histologically proven (neoplastic) tissue remains after resection. This hinders reliable comparisons of IRRs across different resection devices, techniques, endoscopists, and institutions for research or quality evaluation purposes.

Protocols to guide standardized assessment of complete polyp resections could potentially aid in optimizing general polypectomy quality and facilitate more robust evaluation and comparison of IRRs. To inform development of such protocols, an overview of available methods is required. Therefore, we conducted a systematic scoping review to identify the available methods that could aid endoscopists in improving accuracy of visual confirmation of a complete resection, as well as methods that allow IRR measurement based on a histopathological reference standard. This review specifically focusses on polyps resected using regular resection techniques (biopsy forceps, cold snare, or hot snare) and endoscopic mucosal resection (EMR). Findings from this review were used to appraise the feasibility of identified methods to support optimization of general resection quality, and for measuring IRRs. Additionally, we aimed to assess the variability in application of available methods and the comparability of IRRs measured using divergent approaches.

## Methods

This review was conducted according to the Joanna Briggs Institute framework for systematic scoping reviews 9 and was reported according to the Preferred Reporting Items for Systematic Reviews and Meta-Analyses extension for Scoping Reviews (PRIMSA-ScR) checklist (see **Appendix 1s** in the online-only Supplementary Material) 10 . As this is a scoping review, it was not pre-registered with PROSPERO. The research objectives were formulated using the Population(s), Intervention, Comparison, Outcomes, Timing, Study designs (PICOTS) model ( **Table 1s** ).

### Eligibility criteria

Studies evaluating or validating methods aimed at improving the accuracy of visual confirmation of a complete resection, as well as those employing histopathological reference standards to assess resection completeness, were considered eligible. Studies involving polyps resected using regular resection techniques (using either a biopsy forceps, cold snare, or hot snare) or EMR were included. No selection was made based on resection approaches (en bloc vs. piecemeal) or size of included polyps.

Studies using (segmental) polyp recurrence during follow-up colonoscopies 11 or routine histopathological assessment of resection specimen margins (i.e. complete [R0] vs. incomplete [R1] resection 12 ) to determine completeness of resections were excluded, unless these methods explicitly served as a reference standard for evaluation of other methods. The primary reason for omitting these methods was that follow-up colonoscopies and histopathological margin assessment are generally incorporated into routine clinical care. Accordingly, the number of studies adhering to these approaches are numerous and would result in a disproportionate number of included studies.

Secondary reasons for excluding these methods relate to various potential sources of bias adherent to the use of these methods as a reference standard for calculation of IRRs. Primarily, polyp recurrence is dependent on intervals between colonoscopies and only provides long-term (historical) insights into resection quality. Besides, follow-up procedures are only performed in a minority of patients in whom polyps have been previously resected. Moreover, detecting unmarked polypectomy scars at follow-up colonoscopies may prove challenging, as well as distinguishing recurrent from novel neoplasia.

The main source of bias associated with histopathological margin assessment relates to the fact that adequate margin assessment is estimated to be feasible in only 26%–59% of polyps 13 14 15 . This is primarily attributable to factors such as cautery-induced tissue damage and difficulties in margin identification resulting from piecemeal resection, specimen fragmentation, or inadequate specimen orientation (e.g. margins not marked). Besides, although also (partly) applicable to other methods involving a histopathological reference standard, outcomes of histopathological margin assessment may vary depending on the definition used for complete resection 16 , the method used for sectioning of resection specimens 17 , and interobserver variability between pathologists 13 14 .

Studies were also excluded if they were not peer reviewed, did not report original research, were published as conference abstracts, were written in a language other than English, or lacked full-text availability.

### Search strategy and study selection process

A systematic literature search was conducted in the MEDLINE (PubMed), Embase, Web of Science, and Cochrane Library databases. The databases were searched from the inception of the databases up to and including 30 July 2024. A medical librarian assisted in the design of the search strategy and extraction of identified records from the different databases ( **Appendix 2s** ).

All records were imported into Rayyan systematic review software (Cambridge, Massachusetts, USA) 18 . Two authors (Q.N.E.B. and S.Y.) independently screened all records for eligibility. Screening was primarily based on title and abstract, followed by full-text screening. Any conflicts were resolved through discussion.

### Data extraction, data analysis, and appraisal of strengths and limitations

Two authors (Q.N.E.B. and S.Y.) extracted relevant data using structured tables. Extracted data included: 1) study characteristics (author, year of publication, region); 2) method(s) and reference standard(s) used to assess completeness of resections; and 3) outcome data (e.g. IRRs). Extracted data were summarized using descriptive statistics.

## Results

**Fig. 1:**
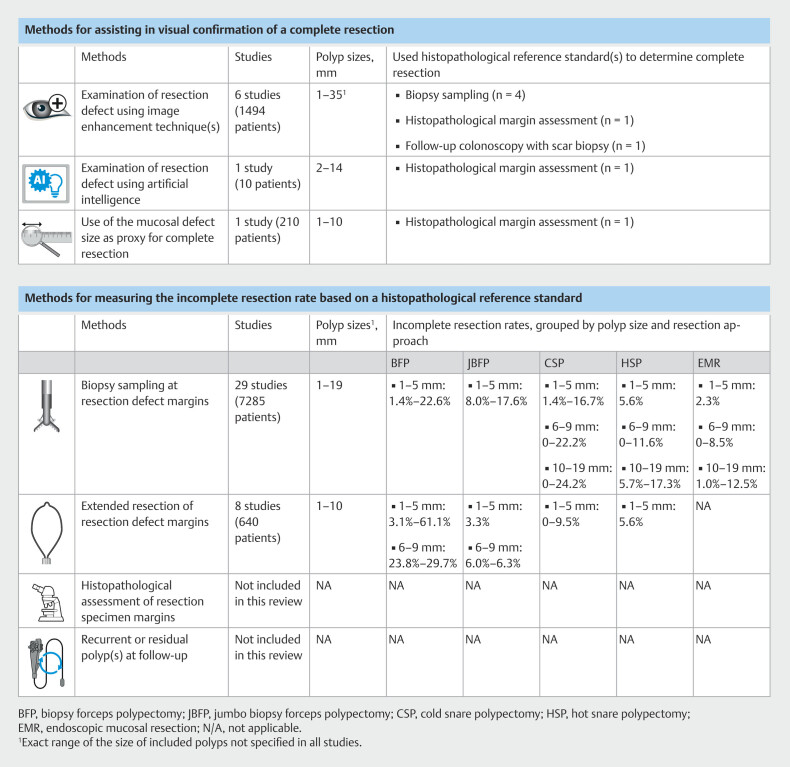
Overview of available approaches to assessing the completeness of polyp resections in clinical practice.

After removing duplicates, our initial search resulted in 4697 unique studies. Based on title and abstract screening, 97 of these studies were considered eligible for full-text review. A total of 56 studies were excluded after full-text review, while 4 additional studies were identified through reference screening. This resulted in the inclusion of 45 studies. The study screening and selection process is summarized in **Fig. 1s** . The majority of included studies were conducted in Asia (44%), followed by Northern America (24%), Europe (22%), and Oceania (8.9%) ( **Table 2s** ).

We identified various methods that could potentially assist in improving accuracy of visual confirmation of a complete resection, as well as methods for measuring IRRs based on a histopathological reference standard. A summary of identified methods and studies is available in Fig. 1 .

### Visual confirmation of complete resection

#### Image enhancement techniques

**Fig. 2:**
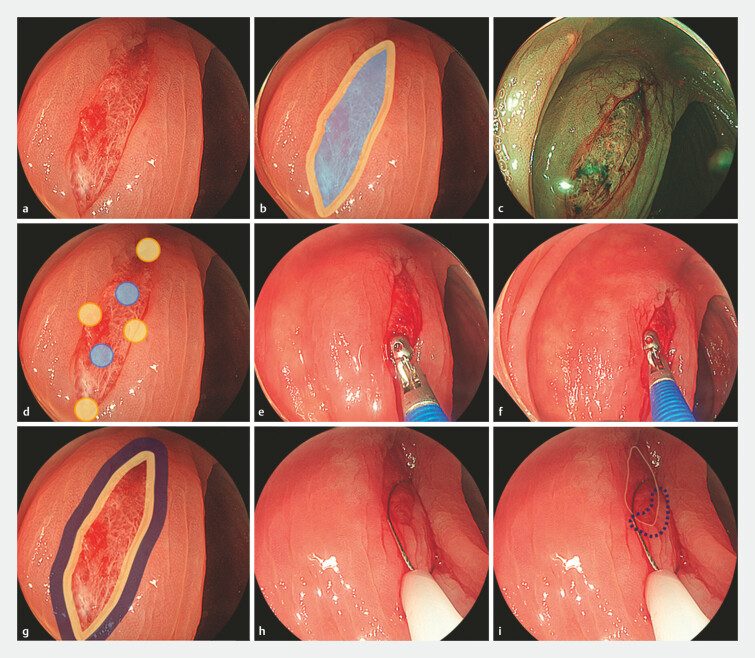
Endoscopy images highlighting the different parts of a polyp resection defect and illustrating methods that may be used for assessment of completeness of resection. **a** Polyp resection defect examined using white-light endoscopy. **b** Polyp resection defect with the lateral margins marked in yellow and the basal margins marked in blue. **c** Polyp resection defect examined using virtual chromoendoscopy (narrow-band imaging). **d** Polyp resection defect with yellow points indicating examples of locations at which lateral margin biopsies may be taken, and blue points indicating examples of locations at which basal margin biopsies may be taken. **e** Biopsy sampling at the lateral margin of the resection defect. **f** Biopsy sampling at the basal margin of the resection defect. **g** Polyp resection defect with the lateral margin marked in yellow and the adjacent rim of normal-appearing mucosa surrounding the resection defect marked in purple. **h** Extended resection of the resection defect margins. **i** Extended resection of the resection defect margins with the lateral margin of the resection defect marked with the yellow line and the surrounding rim of normal-appearing mucosa (included in the illustrated snare grab) marked with the dotted purple line.

Six studies (1494 patients) specifically assessed the potential benefits of the use of image enhancement techniques ( Fig. 2 ) to detect residual polyp tissue at resection defects ( Table 1 ) 19 20 21 22 23 24 . Available studies evaluated the use of chromoendoscopy 23 , chromoendoscopy with magnification endoscopy 19 20 , or virtual chromoendoscopy (VCE) 21 22 24 . Two studies compared endoscopists’ abilities to detect residual polyp tissue using both white-light endoscopy (WLE) and image enhancement techniques 23 24 . One of these studies reported significant enhancement of the endoscopists’ ability to detect residual polyp tissue when using chromoendoscopy 23 . Meanwhile, the other study reported no significant benefits of the use of VCE in terms of residual tissue detection, although a tendency toward a lower IRR was observed using VCE 24 . Other studies reported high accuracies (93%–95%) for detection of residual polyp tissue using chromoendoscopy with magnification endoscopy 19 20 , as well as a relatively low IRR (2.0%) for chromoendoscopy-guided resections using a jumbo biopsy forceps 22 . Despite forceps-aided polypectomy generally being discouraged due to high IRRs 25 26 , one study reported noninferiority in terms of IRR for chromoendoscopy-guided forceps polypectomy compared with cold snare polypectomy using WLE 21 .

**Table:** **Table 1** Studies evaluating the use of image enhancement techniques to assist in visual confirmation of complete polyp resection.

Author (year) [ref] region	Polyp sizes, mm	No. of polyps and patients	Technique(s)	Imaging modality or modalities	Reference standard	Cleansing of resection defect	Results
Hurlstone et al. (2004) 19 Europe	1–35	684 polyps (602 patients)	EMR (not further defined)	CE and ME	Histopathological assessment of resection specimen margins	Yes (saline solution)	Accuracy for detecting residual polyp tissue: 93% (lateral margins), 95% (basal margins) Sensitivity for detecting residual polyp tissue: 79% (lateral margins), 80% (basal margins)
Cipolletta et al. (2009) 20 Europe	≥20	77 polyps (76 patients)	HS-EMR	CE and ME	Multiple margin biopsies (not further specified) and follow-up colonoscopy	Yes (water)	Accuracy for detecting residual polyp tissue: 95% Sensitivity for detecting residual polyp tissue: 98%
Park et al. (2016) 21 Asia	≤5	231 polyps (146 patients)	BFP, CSP	BFP with VCE vs. CSP with WLE	One biopsy at the lateral margin and one biopsy at the basal margin	Yes (saline or epinephrine solution)	IRR: 9.5% vs. 7.5%
Kuwai et al. (2019) 22 Asia	≤5	955 polyps (471 patients)	JBFP	VCE	Follow-up colonoscopy with scar biopsy	Not specified	IRR: 2.0%
O’Morain et al. (2020) 23 Europe	Mostly <10	86 polyps (61 patients)	CSP, HSP	WLE vs. CE	Two basal margin biopsies	Yes (saline solution)	Residual polyp tissue suspected: 4.9% vs. 26.0% Confirmed incomplete resection: 19.8% vs. 15.1%
Jung et al. (2021) 24 Asia	Various (including ≥10)	145 polyps (138 patients)	HS-EMR	WLE vs. VCE	Four lateral margin biopsies (quadrants)	Yes (water)	Residual polyp tissue suspected: 15.4% vs. 12.9% Confirmed incomplete resection: 10.8% vs. 6.5%
BFP, biopsy forceps polypectomy; CE, chromoendoscopy; CSP, cold snare polypectomy; EMR, endoscopic mucosal resection; HS-EMR, hot snare endoscopic mucosal resection; HSP, hot snare polypectomy; IRR, incomplete resection rate; JBFP, jumbo biopsy forceps polypectomy; ME, magnification endoscopy; WLE, white-light endoscopy; VCE, virtual chromoendoscopy.							

Beyond the primary scope of this review, we also identified six studies (915 patients) evaluating the use of image enhancement techniques for detection of recurrent or residual polyp tissue at polypectomy scars during follow-up colonoscopies ( **Table 3s** ) 27 28 29 30 31 32 . All studies comparing detection of residual polyp tissue using WLE with endoscopy using VCE showed superior performance with VCE, especially in terms of sensitivity (differences up to 27%) 28 29 30 31 32 . Moreover, the use of VCE was reported to improve the overall polyp detection rate during surveillance colonoscopies, while 63% of polyps also appeared more extensive when examined using VCE compared with WLE 33 .

#### Other methods

One study (10 patients) evaluated the ability of an artificial intelligence (AI) system trained for polyp characterization (i.e. distinguishing neoplastic from hyperplastic polyps) to assist in detection of residual polyp tissue at resection defects. Accuracy was poor: despite histologically confirmed R0 resections, the system indicated presence of residual neoplastic polyp tissue in all cases 34 . Another study (201 patients) proposed the use of the size of polyp resection defects as a potential surrogate for completeness of resection, suggesting that for polyps ≤10 mm resected using a cold snare, a defect size ≥7 mm predicts complete resection 35 . However, as this study was conducted using retrospectively collected data, prospective evaluation is required to further evaluate the extent to which aiming for a minimum resection defect diameter does allow for preventing incomplete resection ( **Table 4s** ).

### Measurement of IRRs based on a histopathological reference standard

#### Biopsy sampling

A total of 29 studies (7285 patients) described biopsy sampling at resection defect margins ( Fig. 2 ) to measure IRRs ( Table 2 ) 13 21 24 36 37 38 39 40 41 42 43 44 45 46 47 48 49 50 51 52 53 54 55 56 57 58 59 60 61 . All but two studies (27/29, 93%) 44 52 specified that (meticulous) visual inspection of resection defects to detect (and remove) residual polyp tissue was performed prior to biopsy sampling. For visual inspection of the resection defects, studies reported the use of either WLE 41 , VCE 48 61 , or multiple imaging modalities (including magnification endoscopy) 21 24 38 39 40 43 45 46 49 51 53 55 58 59 60 . A total of 14 studies (48%) also specifically reported cleansing (rinsing) of resection defects prior to visual inspection 21 24 37 40 42 43 45 47 48 49 51 53 54 60 .

**Table:** **Table 2** Studies describing biopsy sampling at the resection defect for measurement of the incomplete resection rate.

Author (year) [ref] Region	Polyp sizes, mm	No. of polyps and patients	Technique(s)	No. of biopsies	Location of biopsy sampling	Cleansing of resection defect prior to biopsy sampling	Visual inspection of resection defect prior to biopsy sampling	IRR, % ^1^
Draganov et al. (2012) 36 Northern America	≤6	305 (140 patients)	BFP, JBFP	2	Not specified	Not specified	Yes (not further specified)	17.6–22.6
Liu et al. (2012) 37 Northern America	2–13	65 polyps (47 patients)	BFP, JBFP, CSP, HSP	Not specified	Basal margin	Yes (water)	Yes (not further specified)	9.3–27.3
Pohl et al. (2013) 38 Northern America	5–9	346 polyps (269 patients)	HSP	2	Lateral margin (opposite sites)	Not specified	Yes (WLE, VCE at discretion)	10.1
	10–20			4	Lateral margin (quadrants)			
Park et al. (2016) 21 Asia	≤5	231 polyps (146 patients)	BFP, CSP	2	Lateral margin (one biopsy) and basal margin (one biopsy)	Yes (saline or epinephrine solution)	Yes (BFP: VCE; CSP: WLE)	7.0–9.5
Dwyer et al. (2017) 39 Australia	≤10	299 polyps (181 patients)	CSP (multiple types)	4	Lateral margin (quadrants)	Not specified	Yes (WLE and VCE)	2.6–4.6
Kawamura et al. (2018) 40 Asia	4–9	796 polyps (538 patients)	CSP, HSP	2	Lateral margin (left and right)	Yes (not further specified)	Yes (with magnification and image enhancement at discretion)	1.8–2.6
Kim et al. (2018) 41 Asia	5–9	382 polyps (269 patients)	HSP, CS-EMR	4	Lateral margin (quadrants)	Not specified	Yes (WLE)	7.2–11.6
Maruoka et al. (2018) 42 Asia	≤9	126 polyps (39 patients)	CSP	1	Basal margin ^2^	Yes (water)	Yes (not further specified)	1.0
Papastergiou et al. (2018) 43 Europe	6–10	164 polyps (155 patients)	CS-EMR, HS-EMR	5	Lateral margin (four biopsies, quadrants) and basal margin (one biopsy)	Yes (water)	Yes (WLE, VCE at discretion)	3.7–7.2
Zhang et al. (2018) 44 Asia	6–9	525 polyps (358 patients)	CSP, HS-EMR	5	Lateral margin (four biopsies, quadrants) and basal margin (one biopsy)	Not specified	Not specified	1.5–8.5
Huh et al. (2019) 45 Asia	≤5	196 polyps (169 patients)	JBFP, CSP	2	Lateral margin (not further specified)	Yes (saline solution)	Yes (WLE and VCE)	7.8–8.0
Caliţa et al. (2020) 46 Europe	Various (including ≥20 mm)	326 polyps (210 patients)	CSP, BFP, HSP	Not specified	Lateral margin (not further specified)	Not specified	Yes (WLE and VCE)	12.0–16.7
Desai et al. (2020) 47 Northern America	≤6	261 polyps (151 patients)	JBFP, CSP	1–3	Basal margin (1–3 biopsies)	Yes (not further specified)	Yes (not further specified)	7.7–11.1
Li et al. (2020) 48 Asia	6–9	763 polyps (404 patients)	CSP, CS-EMR, HS-EMR	3	Lateral margin (two biopsies) and basal margin (one biopsy)	Yes (not further specified)	Yes (VCE)	4.5–18.4
	10–20			5	Lateral margin (four biopsies, quadrants) and basal margin (one biopsy)			
Jung et al. (2021) 24 Asia	Various (including ≥10)	145 polyps (138 patients)	HS-EMR	4	Lateral margin (four biopsies, quadrants)	Yes (water)	Yes (WLE or VCE)	6.5–10.8 ^3^
Park et al. (2021) 13 Northern America	≤5	92 polyps (234 patients)	CSP, HSP	2	Lateral margin (not further specified)	Not specified	Yes (not further specified)	9.3–10.9
	6–20			4	Lateral margin (quadrants)			
Pedersen et al. (2021) 49 Europe	<10	327 polyps (246 patients)	CSP, HSP	2	Lateral margin (not further specified)	Yes (not further specified)	Yes (WLE and VCE)	13.4–17.4
	≥10			4	Lateral margin (not further specified)			
De Benito Sanz et al. (2022) 50 Europe	5–9	791 polyps (496 patients)	CSP, HSP	2	Lateral margin (left and right) and targeted biopsies of mucosa with suspicious appearance	Not specified	Yes (not further specified)	6.0–7.5
Ma et al. (2022) 51 Asia	5–9	440 polyps (261 patients)	CSP	2	Lateral margin (left and right)	Yes (water)	Yes (VCE or magnification)	2.3
	10–15			4	Lateral margin (quadrants)			
Meng et al. (2022) 52 Asia	4–9	301 polyps (249 patients)	CSP, HSP	Not specified	Lateral margin and basal margin (not further specified)	Not specified	Not specified	6.6–5.5
Pedersen et al. (2022) 53 Europe	4–6	601 polyps (425 patients)	CSP, HSP	2	Lateral margin (not further specified)	Yes (water)	Yes (WLE, VCE if available)	7.4–10.7
	7–9			3	Lateral margin (not further specified)			
Perrod et al. (2022) 54 Europe	≤3	121 polyps (123 patients)	BFP, CSP	≥2	Lateral margin (not further specified)	Yes (saline solution)	Yes (not further specified)	6.7–9.2
Rex et al. (2022) 55 Northern America	6–15	286 polyps (235 patients)	CSP, CS-EMR, HSP, HS-EMR	4	Lateral margin (four biopsies, quadrants) and basal margin (one biopsy)	Not specified	Yes (WLE, VCE at discretion)	0–6.2
Wei et al. (2022) 56 Northern America	≤3	279 polyps (179 patients)	BFP, CSP	2	Lateral margin (not further specified)	Not specified	Yes (not further specified)	1.4–1.7
Wei et al. (2022) 57 Northern America	4–9	291 polyps (159 patients)	CSP (multiple types)	2	Lateral margin (not further specified)	Not specified	Yes (not further specified)	1.4–2.8
Mangira et al. (2023) 58 Australia	10–19	350 polyps (295 patients)	CSP, CS-EMR	Other	En bloc resections: lateral margin (four biopsies); piecemeal resections: lateral margin (eight biopsies and at areas of snare overlap)	Not specified	Yes (WLE and VCE)	0.3–1.7
Motchum et al. (2023) 59 Northern America	4–9	204 polyps (429 patients)	CS-EMR	2	Lateral margin (opposite sides)	Not specified	Yes (WLE and VCE)	1.6
	10–20			4	Lateral margin (quadrants)			
Kim et al. (2023) 60 Asia	6–10	444 polyps (327 patients)	CSP, CS-EMR	2	Lateral margin (not further specified)	Yes (saline solution)	Yes (WLE and VCE)	8.1–10.2
Von Renteln et al. (2023) 61 Northern America	4–9	182 polyps (413 patients)	CSP	2	Lateral margin (top and bottom)	Not specified	Yes (VCE)	18.8
	10–20			4	Lateral margin (quadrants)			
BFP, biopsy forceps polypectomy; CS-EMR, cold snare endoscopic mucosal resection; CSP, cold snare polypectomy; HS-EMR, hot snare endoscopic mucosal resection; HSP, hot snare polypectomy; IRR, incomplete resection rate; JBFP, jumbo biopsy forceps polypectomy; VCE, virtual chromoendoscopy; WLE, white-light endoscopy ^1^ Reported ranges represent the range of IRRs as reported for different resection devices and techniques, not accounting for factors such as polyp size or (suspected) histological polyp subtype. ^2^ Biopsy from the base of the polypectomy scar taken at follow-up colonoscopy 3 weeks after the primary procedure. ^3^ IRR differed based on used imaging modality: 6.5% for white-light endoscopy, 10.8% for narrow-band imaging.								

With regard to both the location and number of biopsies that were taken, the identified studies used divergent approaches. In all but one study, the location at which biopsy sampling was performed was specified: biopsies were taken at the lateral resection margin (19/28 studies, 68%), the basal resection margin (3/28 studies, 11%), or both (6/28 studies, 21%) ( Table 3 ). One study described additional targeted biopsy sampling at areas with suspected (residual) neoplastic tissue 50 , while another study described additional biopsy sampling at areas of snare overlap for piecemeal resections 58 . In terms of the number of biopsies, the (minimum) number of biopsies varied between one and eight across the different studies 13 21 24 36 38 39 40 41 42 43 44 45 47 48 49 50 51 53 54 55 56 57 58 59 60 61 . However, not all studies described a standardized number of biopsies 37 46 52 . Some studies adhered to different biopsy protocols for polyps in different size categories 13 38 48 49 51 58 59 61 .

**Fig. 3:**
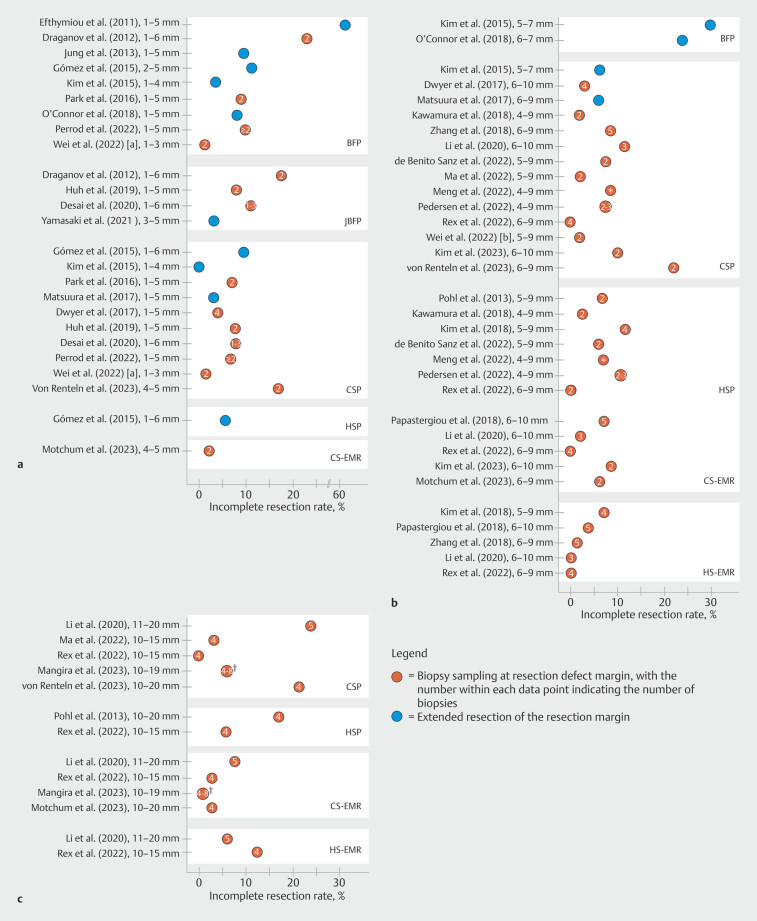
Overview of incomplete resection rates as reported across studies, measured using divergent approaches. Polyps are grouped by polyp size and resection device and technique. **a** Polyps ~1–5 mm. **b** Polyps ~6–9 mm. **c** Polyps ~10–19–mm. BFP, biopsy forceps polypectomy; CS-EMR, cold snare endoscopic mucosal resection; CSP, cold snare polypectomy; HS-EMR, hot snare endoscopic mucosal resection; HSP, hot snare polypectomy; JBFP, jumbo biopsy forceps polypectomy. *Unspecified number of biopsies; ^†^ En bloc resections: 4 biopsies, piecemeal resections: 8 biopsies and at areas of snare overlap.

Reported IRRs ranged between 0% and 24.2% 13 21 24 36 37 38 39 40 41 42 43 44 45 46 47 48 49 50 51 52 53 54 55 56 57 58 59 60 61 . The range of reported IRRs, grouped by polyp size category and resection device and technique, is reported in Fig. 1 . Studies in which IRRs were reported grouped by size (category) and resection device and technique are included in Fig. 3 and **Tables 5s–7s** , illustrating the variability in IRRs across different studies.

**Table:** **Table 3** Studies describing extended resection of the resection defect to measure the incomplete resection rate.

Author (year) [ref] Region	Polyp sizes (mm)	Number of polyps and patients	Technique(s)	Extended resection approach	Cleansing of resection defect prior to extended resection	Visual inspection of resection defect prior to extended resection	IRR, % ^1^
Efthymiou et al. (2011) 62 Australia	≤5	54 polyps (52 patients)	BFP	HS-EMR: lifting (saline solution) and extended resection with a 1–2-mm margin	Yes (water)	Yes (not further specified)	61.1
Jung et al. (2013) 63 Asia	≤5	88 polyps (65 patients)	BFP	HS-EMR: lifting (saline, indigo carmine, epinephrine solution) and extended resection with a 1–3-mm margin	Yes (saline solution)	Yes (CE)	9.3
Gómez et al. (2015) 64 Northern America	2–5	62 polyps (60 patients)	CSP, HSP, BFP	CS-EMR: lifting (saline solution) and extended resection with a 1–3-mm margin; if extended resection unsuccessful: ≥4 margin biopsies	Not specified	Not specified	9
Kim et al. (2015) 65 Asia	≤7	145 polyps (128 patients)	CSP, BFP	HS-EMR: lifting (saline, epinephrine solution) and extended resection with a 1–2-mm margin	Not specified	Not specified	3.4–17.4
Matsuura et al. (2017) 14 Asia	≤9	307 polyps (120 patients)	CSP	CS-EMR: lifting (saline solution) and extended resection with a 1–3-mm margin	Not specified	Not specified	3.9
O’Connor et al. (2018) 66 Australia	≤7	60 polyps (42 patients)	BFP	CS-EMR: lifting (saline, indigo carmine, epinephrine solution) and extended resection with a 1–2-mm margin	Not specified	Yes (VCE and ME at discretion)	14
Yamasaki et al. (2021) 67 Asia	≤5	120 polyps (80 patients)	JBFP	HS-EMR: lifting (saline solution) and extended resection with a 2–3-mm margin	Yes (water)	Yes (VCE or CE)	3.3
Lu et al. (2023) 68 Asia	Mostly <10	93 polyps (93 patients)	BFP, CSP, HSP, EMR	CS-EMR: lifting (saline solution) and extended resection (no margin described)	Not specified	Not specified	4.2–8.9
BFP, biopsy forceps polypectomy; CE, chromoendoscopy; CS-EMR, cold snare endoscopic mucosal resection; CSP, cold snare polypectomy; EMR, endoscopic mucosal resection; HS-EMR, hot snare endoscopic mucosal resection; HSP, hot snare polypectomy; IRR, incomplete resection rate; JBFP, jumbo biopsy forceps polypectomy; ME, magnification endoscopy; VCE, virtual chromoendoscopy. ^1^ Reported ranges represent the range of IRRs as reported for different resection devices and techniques, not accounting for factors such as polyp size or (suspected) histological polyp subtype.							

### Extended margin resection

Eight studies (640 patients) performed resections of normal-appearing mucosa surrounding resection defects (lateral margins) ( Fig. 2 ) to measure IRRs 14 62 63 64 65 66 67 68 ( Table 3 ). Four of these studies specifically reported (meticulous) visual inspection of resection defects, mostly using (virtual) chromoendoscopy, prior to performing extended resections 62 63 66 67 . Three studies specifically reported cleansing (rinsing) of resection defects prior to visual inspection 62 63 67 .

Extended resections were performed by either cold snare 14 64 66 68 or hot snare 62 63 65 67 EMR. The extent (width) of the extended resection varied, with studies describing either an additional 1–2 mm 62 65 66 , 1–3 mm 14 63 64 , or 2–3 mm 67 resection. One study did not report a specific (minimum) extent of the extended resection 68 .

Reported IRRs ranged between 3.3% and 61.1% 14 62 63 64 65 66 67 68 . The range of reported IRRs, grouped by polyp size category and resection device and technique, is reported in Fig. 1 . Studies in which IRRs were reported grouped by size (category) and resection method are included in Fig. 1 and **Tables 5s–7s** , illustrating the variability in IRRs across different studies.

## Discussion

This review provides a comprehensive overview of the available approaches to assessing completeness of colorectal polyp resections. The summarized results emphasize that incomplete resection represents an important clinical challenge: IRRs exceeding 10% were observed across polyps of all size categories and for almost all resection devices and techniques. Evidence regarding the best approach for assessing incomplete resection in clinical practice is limited. Meanwhile, some studies suggest that the use of image enhancement techniques could aid in detection of residual polyp tissue 19 20 21 22 23 27 28 29 30 31 32 . For measurement of IRRs based on a histopathological reference standard, studies employed divergent biopsy sampling and extended margin resection approaches. Approaches differed in terms of factors such as the location (lateral margin, basal margin, or both) and number of biopsies (1–8), and the extent (width) of the extended resections (1–3 mm). Of note, IRRs varied considerably across studies, ranging from 0% to 24.2% in studies that used biopsy sampling, and from 0% to 61.1% in studies that used extended margin resection.

The risk for incomplete polyp resection depends on polyp characteristics, resection device and technique, and endoscopist skill. Larger polyp size increases the risk of incomplete resection 8 38 48 65 69 , likely because achieving an adequate margin becomes more challenging and piecemeal removal is more often required. Piecemeal resections, in turn, are associated with higher IRRs than en bloc resections 44 48 50 61 70 , a difference that may be attributable to residual polyp tissue (“tissue bridges”) remaining between sequential snare captures during piecemeal removal 71 . Histological subtype also influences the risk for incomplete resection, with various studies reporting higher IRRs for sessile serrated lesions compared with adenomatous polyps 38 39 41 48 49 50 51 53 59 65 . Additionally, a polyp location in the proximal colon 49 62 and flat polyp morphology 50 61 have been linked to higher IRRs. Regarding resection device and technique, biopsy forceps polypectomy is discouraged due to the associated relatively high risk for incomplete resection, particularly for polyps >3 mm 25 26 . Depending on polyp size and morphology, specific (advanced) resection techniques and (add-on) devices may help to reduce IRRs 59 72 73 74 75 76 77 . Finally, the significant variation in IRRs that exists among endoscopists 38 49 50 59 may be explained by differences in endoscopists’ skills and proficiency in specific techniques 78 , as well as variations in the quality of resection defect assessment (e.g. use of image enhancement techniques, duration of inspection, and attention to detail).

This review offers several valuable insights for endoscopists in daily practice. First, it emphasizes that incomplete polyp resection remains common for any polyp size category – diminutive (≤5 mm), small (6–9 mm), and large (≥10 mm). Reported IRRs exceeded 10%, and ranged up to over 60%, in some studies ( Fig. 1 , Fig. 3 , **Tables 5s–7s** ). As such, clinicians must remain vigilant about incomplete resection even in smaller and seemingly less harmful polyps. Given that patients with only a few low-risk lesions often do not undergo follow-up for up to 10 years according to current surveillance guidelines 79 80 , any incomplete polyp resection may contribute to an increased risk of interval colorectal cancer.

Second, this review corroborates findings of previous studies illustrating relatively high IRRs for resections performed using (jumbo) biopsy forceps ( Fig. 1 , Fig. 3 , **Table 5s, Table 6s** ). This reinforces the importance of following European and American guidelines, which recommend avoiding biopsy forceps polypectomy, particularly for polyps >3 mm 25 26 . To optimize resection quality, this recommendation should be complied with in addition to other key practice recommendations, such as aiming for en bloc resection over piecemeal resection whenever possible, and ensuring a clear 1–2-mm margin for cold snare polypectomies of lesions <10 mm.

Third, the IRRs reported in this review primarily reflect those observed in study settings, where meticulous visual inspection of resection defects is typically conducted before tissue sampling to measure IRRs. In contrast, in routine clinical practice, visual inspection is likely to be less consistently and thoroughly performed. Accordingly, IRRs in daily practice settings are likely to even exceed those outlined in this review. This provides an additional argument for clinicians to be encouraged to perform standardized and meticulous visual inspection after any polyp resection, particularly as associated time-related burdens of visual inspection are relatively low and most modern endoscopy processors allow for the use of electronic image enhancement techniques with a single push of a button.

Given the approach for visual defect inspection described in most studies evaluating IRRs, inspection should at least involve thorough cleansing of the resection defect, followed by meticulous inspection using high-definition WLE 13 14 21 24 36 37 38 39 40 41 42 43 44 45 46 47 48 49 50 51 52 53 54 55 56 57 58 59 60 61 62 63 64 65 66 67 68 . While evidence is limited, the use of image enhancement techniques may help improve the accuracy of residual polyp tissue detection 19 20 21 22 23 27 28 29 30 31 32 . Active bleeding or the presence of clips may hinder adequate visual inspection, and in these cases, additional measures such as targeted biopsies may be necessary to assess resection completeness 50 . Standardized post-resection photo documentation for all polyps may represent an additional measure to ensure that resection defects are appropriately cleaned and visualized, and should therefore, despite a paucity of supporting data, also be encouraged.

Most current society guidelines lack recommendations for (standardized) post-resection inspection and photo documentation for polyps <20 mm 25 26 81 . This may contribute to the fact that, in daily practice, consistent examination of resection sites is unlikely to be uniformly applied across all polyps, endoscopists, and institutions. Accordingly, efforts to establish recommendations for standardized visual inspection seem warranted, particularly as standardized imaging protocols have previously been shown to enhance endoscopist performance and reduce interobserver variability 82 83 . Considering the limited available evidence, such recommendations may need to be primarily based on expert consensus.

In the future, AI may emerge as an additional diagnostic tool to assist endoscopists in examining resection defects. However, as outlined in this review, algorithms specifically trained to detect residual polyp tissue at resection defects are currently lacking. This may partly relate to the absence of a robust, universally accepted reference standard for complete polyp resection. Additionally, since recurrent polyps may arise from a few microscopic abnormal crypts rather than visible (macroscopic) polyp tissue, it remains uncertain whether AI can ultimately surpass the human eye in detecting residual tissue. Nevertheless, AI systems could potentially also be trained to provide active feedback on aspects such as the adequacy of resection defect cleansing, inspection, and photo documentation. Accordingly, further exploration of the use of AI for optimizing polypectomy quality should be pursued.

To measure IRRs for research or quality evaluation purposes, methods that allow for histopathological examination of the presence of residual (microscopic) polyp tissue are required. Among the methods discussed in this review, biopsy sampling is the most commonly used approach for this purpose ( Table 2 , Table 3 , Fig. 3 ). This is likely due to two main factors. First, biopsy sampling is associated with lower procedural burdens compared with extended resections, as extended resection typically requires both lifting and multiple resections of mucosa adjacent to the defect. Second, biopsy sampling may be more feasible for larger polyps: studies using biopsy sampling included polyps ≤20 mm, whereas those involving extended resections mostly only included polyps <10 mm.

As extended resections generally provide more tissue than biopsy sampling, extended resections could be hypothesized to yield a higher sensitivity for detecting residual polyp tissue and, therefore, result in higher IRRs. However, this review suggests that, for polyps within similar categories, IRRs measured by extended resection are not consistently higher than those measured by biopsy sampling ( Fig. 3 , **Tables 5s–7s** ). Moreover, at first glance, IRRs do not appear to be directly related to the number of biopsies taken. These results should, however, be interpreted with caution as we did not perform formal statistical analyses and were unable to further stratify results based on factors such as histological subtype, endoscopist experience, or the location of biopsy sampling. Future studies comparing different IRR measurement methods, with stratification for key risk factors for incomplete resection, are needed to provide more insight into the diagnostic value of the different approaches.

While the optimal approach to measuring IRRs is still uncertain, adoption of a standardized IRR measurement approach is needed to diminish the chosen method as a potential source of bias when evaluating or comparing IRRs. Moreover, correlating IRRs measured using a standardized approach with clinical outcomes may help to establish the IRR as a relevant performance measure for colonoscopy. Given the heterogeneity of available evidence, similarly to a protocol for standardized visual inspection, a protocol for standardized IRR measurement may preferably be established through expert consensus initiatives.

This review has several strengths. Most notably, it is the first to provide a comprehensive overview of the methods available for assessing the completeness of colorectal polyp resections, while also highlighting variability in their application and comparability of IRRs measured using different approaches. Furthermore, it offers additional insights into the extent of incomplete resections across various polyp categories and resection devices and techniques. Finally, the literature search and screening process was systematically performed by two independent authors.

A limitation of this study relates to the exclusion of studies using (segmental) polyp recurrence or histopathological resection specimen margin assessment (R0 vs. R1 resection) 15 as the primary outcome measure used to determine complete resection. The exclusion of these methods was carefully considered based on the reasons outlined in the methods section. However, it should be emphasized that these methods could still provide valuable insights into polypectomy quality. This is particularly true for histopathological margin assessment of polyps with clearly identifiable margins (e.g. en bloc resected polyps where the specimen is intact and the margins are not compromised by cautery artifacts) 13 14 15 . Future studies should aim to compare IRRs determined by histopathological margin assessment with the methods included in this review to better define the complementary role of (routine) histopathological assessment in IRR evaluation.

Another limitation of this study is the decision not to pool data regarding the diagnostic performance of different methods. However, this decision reflects an intentional methodological choice given that the primary aim of this study was to map the existing literature and summarize the range of available evidence. Besides, the IRRs as reported across the studies were generally not stratified for all major risk factors for incomplete resection (i.e. polyp size, resection method, histological subtype, endoscopist experience), thereby complicating a meaningful comparison of IRRs. Nevertheless, the findings of this review may serve as a foundation for future (meta-analytical) analyses. Such analyses should aim to evaluate the impact of the method used for IRR measurement while appropriately accounting for the aforementioned risk factors. This may be achieved, for example, by requesting original or additional source data from studies included in this review.

In conclusion, incomplete polyp resection represents an important clinical challenge across polyps of all size categories and resection devices and techniques. Different methods are available to potentially aid endoscopists in improving the accuracy of visual confirmation of complete polyp resection, as well as measuring IRRs. Recommendations for standardized visual inspection of resection defects to detect residual polyp tissue, utilizing both WLE and image enhancement techniques, may represent the most feasible approach for optimizing general quality of polyp resections at this point. For measurement of IRRs for research or quality evaluation purposes, protocols involving a combination of visual inspection and biopsy sampling may be preferred. The findings of this review should serve as a starting point for expert consensus initiatives to establish protocols for standardized assessment of completeness of colorectal polyp resections.

## Supplementary Material

Supplementary Material Zusatzmaterial
