# Supplementary material for: Approaches to assessing completeness of colorectal polyp resections in clinical practice: a systematic scoping review
Source: Endoscopy. 2026 Feb 13;58(6):627–43. doi: 10.1055/a-2783-3897 (PMC13295129; doi:10.1055/a-2783-3897)
Supplement: Supplementary file 1 — Supplementary Material [file 10-1055-a-2783-3897_28079461.pdf]

SUPPLEMENTARY MATERIALS

Approaches to assessing completeness of colorectal polyp resections in clinical practice: a systematic scoping review

Querijn N. E. van Bokhorst, Silpa Yarra, Manon van der Vlugt, Heiko Pohl, Evelien Dekker, Aasma Shaukat

Table 1s PICOTS table

**P** - Population(s)	Endoscopic resections of colorectal polyps
**I** - Intervention	Methods aimed at improving the accuracy of visual confirmation of a complete resection, as well as those employing a histopathological reference standard to assess resection completeness
**C** - Comparison	None
**O** - Outcomes	▪ Overview of methods used for assessing completeness of polyp resections<br>▪ Insights into variability regarding application of identified methods<br>▪ Insights into diagnostic performance of identified methods
**T** - Timing	From inception of included databases until July 30, 2024
**S** - Study designs	Cohort studies, cross-sectional studies, randomized controlled trials, case series

Table 2s Identified studies stratified by geographical region

	**Asia (%)**	**Europe (%)**	**Northern America (%)**	**Oceania (%)**
Biopsy sampling (n = 29)	11 (38)	6 (21)	10 (34)	2 (7)
Extended resection (n = 8)	5 (63)	0 (0)	1 (13)	2 (25)
Image enhancement techniques (n = 6)	3 (50)	3 (50)	0 (0)	0 (0)
Other (n = 2)	1 (50)	1 (50)	0 (0)	0 (0)
**Total (n = 45)**	**20 (44)**	**10 (22)**	**11 (24)**	**4 (8.9)**

Table 3s Studies evaluating image enhancement techniques for detection of residual or recurrent polyp tissue at resection scars.

Author (year)	Region	Number of scars and patients	Imaging modalities	Reference standard	Accuracy (%)	Sensitivity (%)	NPV (%)
Shahid et al. (2012)[1]	Northern-America	129 scars (92 patients)	▪ NBI or FICE<br>▪ Probe-based confocal laser endomicroscopy	In case of suspected recurrence: targeted biopsies and treatment (resection) of suspected area; in case of no suspected recurrence: four scar biopsies	77<br>81	97*<br>72*	91<br>99
Desomer et al. (2017)[2]	Australia	183 scars (183 patients)	▪ WLE<br>▪ WLE and NBI	In case of suspected recurrence: snare resection; in case of no suspected recurrence: one biopsy per 5 mm	91.3<br>94.0	66.7<br>93.3	93.6<br>98.6
Kandel et al. (2019)[3]	Northern-America	255 scars (235 patients)	▪ WLE<br>▪ WLE-NF<br>▪ NBI<br>▪ NBI-NF<br>▪ WLE (high confidence diagnosis)<br>▪ WLE-NF (high confidence diagnosis)<br>▪ NBI (high confidence diagnosis)<br>▪ NBI-NF (high confidence diagnosis)	In case of suspected recurrence: snare resection or avulsion; in case of no suspected recurrence: two to four biopsies at the edge of the scar	93.7<br>92.9<br>93.7<br>93.7<br>96.7<br>97.0<br>96.7<br>96.6	90.3<br>91.9<br>91.9<br>93.5<br>93.6<br>95.7<br>95.7<br>100	96.8<br>97.3<br>97.3<br>97.8<br>98.1<br>98.8<br>98.8<br>100
Pu et al. (2020)[4]	Australia	100 scars (82 patients)	▪ WLE<br>▪ NBI<br>▪ WLE (high confidence diagnosis)<br>▪ NBI (high confidence diagnosis)	Scar biopsy sampling (not further specified)	93.0<br>95.0<br>94.7<br>100	73.7*<br>100*<br>73.7*<br>100*	94.1<br>100<br>93.8<br>100
Joao et al. (2023)[5]	Europe	203 scars (194 patients)	▪ WLE<br>▪ NBI	In case of suspected recurrence: targeted biopsies and treatment (resection) of the suspected area, as well as two biopsies at the edge of the scar; in case of no suspected recurrence: two scar biopsies (not further specified)	93.6<br>94.6	83.3*<br>90.0*	93.3<br>95.8
Ortiz et al. (2024)[6]	Europe	173 scars (129 patients)	▪ WLE<br>▪ LCI<br>▪ WLE and BLI<br>▪ LCI and BLI	In case of suspected recurrence: resection of suspected area; in case of no suspected recurrence: two to four biopsies from the center and lateral margins of the scar	90.2<br>91.9<br>90.2<br>92.5	89.3<br>96.4<br>91.1<br>96.4	94.6<br>98.1<br>95.5<br>98.2

NPV, negative predictive value; NBI, narrow-band imaging; FICE, Fujifilm Intelligent Color Enhancement; WLE, white light endoscopy; WLE-NF, white light endoscopy with near focus mode; NBI-NF, narrow-band imaging with near focus mode; LCI, linked color imaging; *Significant difference (p<0.05).

Table 4s Studies evaluating other methods for assisting in visual confirmation of a complete resection.

Author (year)	Region	Polyp sizes (mm)	Number of polyps and patients	Technique(s)	Reference standard	Results
Kliegis et al. (2021)[7]	Europe	2-14	17 polyps (10 patients)	Computer-aided diagnosis system for polyp detection and characterization	Histopathological margin assessment (R0 vs. R1 resection)	All polyps concerned histologically confirmed R0 resections, with the computer-aided diagnosis system indicating presence of remaining neoplastic polyp tissue in all cases (accuracy: 0%)
Kudo et al. (2021)[8]	Asia	≤10	500 polyps (201 patients)	Standardized resection technique to allow for the use of resection defect size as a proxy for complete resection	Histopathological margin assessment (R0 vs. R1 resection)	Complete resection (lateral and basal margins free from neoplastic polyp tissue) with presence of muscularis mucosae was achieved for 417/417 (100%) polyps with a defect size ≥7 mm

Thieme

Table s5 Incomplete resection rate as reported in the literature for polyps ~1-5 mm, grouped by polyp resection device and technique

Author (year)	Size of included polyps, mm	Histological subtype(s) of included polyps	Resection device / technique	Method for assessment of IRR	Number of biopsies	Incompletely resected polyps, n	IRR, %
Efthymiou et al. (2011)[9]	1-5	Adenomas, HPs*	BFP	Additional resection	*NA*	33/54	61.1
Draganov et al. (2012)[10]	1-6	Adenomas*	BFP	Biopsy sampling	2	7/31	22.6
Jung et al. (2013)[11]	1-5	Adenomas, HPs*	BFP	Additional resection	*NA*	8/86	9.3
Gomez et al. (2015)[12]	2-5	Adenomas, SSLs, HPs	BFP	Additional resection	*NA*	2/18	11.1
Kim et al. (2015)[13]	1-4	Adenomas, HPs, other*	BFP	Additional resection	*NA*	1/32	3.1
Park et al. (2016)[14]	1-5	Adenomas, HPs*	BFP	Biopsy sampling	2	11/120	9.2
O'Connor et al. (2018)[15]	1-5	Adenomas, HPs*	BFP	Additional resection	*NA*	3/36	8.3
Perrod et al. (2022)[16]	1-5	Adenomas, SSLs	BFP	Biopsy sampling	≥2	6/61	9.8
Wei et al. (2022)[a][17]	1-3	Adenomas, SSLs, HPs, other	BFP	Biopsy sampling	2	2/141	1.4
Draganov et al. (2012)[10]	1-6	Adenomas*	JBFP	Biopsy sampling	2	6/34	17.6
Huh et al. (2019)[18]	1-5	Adenomas*	JBFP	Biopsy sampling	2	7/87	8.0
Desai et al. (2020)[19]	1-6	Adenomas, SSLs, HPs, other	JBFP	Biopsy sampling	1-3	16/144	11.1
Yamasaki et al. (2021)[20]	3-5	Adenomas, SSLs, HPs	JBFP	Additional resection	*NA*	4/120	3.3
Gómez et al. (2015)[12]	1-6	Adenomas, SSLs, HPs	CSP	Additional resection	*NA*	2/21	9.5
Kim et al. (2015)[13]	1-4	Adenomas, HPs, other*	CSP	Additional resection	*NA*	0/27	0
Park et al. (2016)[14]	1-5	Adenomas, HPs*	CSP	Biopsy sampling	2	8/115	7.0
Matsuura et al. (2017)[21]	1-5	Adenomas, carcinoma(s)	CSP	Additional resection	*NA*	7/223	3.1
Dwyer et al. (2017)[22]	1-5	Adenomas, SSLs, HPs	CSP	Biopsy sampling	4	6/158	3.8
Huh et al. (2019)[18]	1-5	Adenomas, HPs, other*	CSP	Biopsy sampling	2	7/90	7.8
Desai et al. (2020)[19]	1-6	Adenomas, SSLs, HPs, other	CSP	Biopsy sampling	1-3	9/117	7.7
Perrod et al. (2022)[16]	1-5	Adenomas, SSLs	CSP	Biopsy sampling	≥2	4/60	6.7
Wei et al. (2022)[a][17]	1-3	Adenomas, SSLs, HPs, other	CSP	Biopsy sampling	2	2/138	1.4
von Renteln et al. (2023)[23]	4-5	Adenomas, carcinoma(s), SSLs, HPs, other	CSP	Biopsy sampling	2	13/78	16.7
Gomez et al. (2015)[12]	1-6	Adenomas, SSLs, HPs, other	HSP	Additional resection	*NA*	1/18	5.6
Motchum et al. (2023)[24]	4-5	Adenomas, SSLs, HPs	CS-EMR	Biopsy sampling	2	2/87	2.3

IRR, incomplete resection rate; HPs, hyperplastic polyps; BFP, biopsy forceps polypectomy; NA, not applicable; SSLs, sessile serrated lesions; JBFP, jumbo biopsy forceps polypectomy; CSP, cold snare polypectomy; HSP, hot snare polypectomy; CS-EMR, cold snare endoscopic mucosal resection; *Inclusion of SSLs and/or assignment of SSLs to either the adenomas or HPs group unspecified.

Table 6s Incomplete resection rate as reported in the literature for polyps ~6-9 mm, grouped by polyp resection device and technique.

Author (year)	Size of included polyps, mm	Histological subtype(s) of included polyps	Resection device / technique	Method for assessment of IRR	Number of biopsies	Incompletely resected polyps, n	IRR, %
Kim et al. (2015)[13]	5-7	Adenomas, HPs*	BFP	Additional resection	*NA*	11/37	29.7
O'Connor et al. (2018)[15]	6-7	Adenomas, HPs*	BFP	Additional resection	*NA*	5/21	23.8
Kim et al. (2015)[13]	5-7	Adenomas*	CSP	Additional resection	*NA*	2/32	6.3
Dwyer et al. (2017)[22]	6-10	Adenomas, SSLs, HPs	CSP	Biopsy sampling	4	4/141	2.8
Matsuura et al. (2017)[21]	6-9	Adenomas, carcinoma(s)	CSP	Additional resection	*NA*	5/84	6.0
Kawamura et al. (2018)[25]	4-9	Adenomas	CSP	Biopsy sampling	2	6/341	1.8
Zhang et al. (2018)[26]	6-9	Adenomas, SSLs	CSP	Biopsy sampling	5	18/212	8.5
Li et al. (2020)[27]	6-10	Adenomas, carcinoma(s), SSLs	CSP	Biopsy sampling	3	13/112	11.6
de Benito Sanz et al. (2022)[28]	5-9	Adenomas, carcinoma(s), SSLs, HPs, other	CSP	Biopsy sampling	2	29/387	7.5
Ma et al. (2022)[29]	5-9	Adenomas, SSLs	CSP	Biopsy sampling	2	7/353	2.0
Meng et al. (2022)[30]	4-9	Adenomas, SSLs	CSP	Biopsy sampling	Unspecified	13/154	8.4
Pedersen et al. (2022)[31]	4-9	Adenomas, SSLs, HPs, other	CSP	Biopsy sampling	2-3†	21/283	7.4
Rex et al. (2022)[32]	6-9	Adenomas, SSLs, HPs, other	CSP	Biopsy sampling	4	0/41	0
Wei et al. (2022)[b][33]	5-9	Adenomas, SSLs, HPs, other	CSP	Biopsy sampling	2	6/291	2.1
Kim et al. (2023)[34]	6-10	Adenomas, carcinoma(s), SSLs, HPs	CSP	Biopsy sampling	*2*	22/215	10.2
von Renteln et al. (2023)[23]	6-9	Adenomas, carcinoma(s), SSLs, HPs, other	CSP	Biopsy sampling	*2*	8/36	22.2
Pohl et al. (2013)[35]	5-9	Adenomas, carcinoma(s), SSLs	HSP	Biopsy sampling	2	16/236	6.8
Kawamura et al. (2018)[25]	4-9	Adenomas	HSP	Biopsy sampling	2	9/346	2.6
Kim et al. (2018)[36]	5-9	Adenomas, SSLs, HPs	HSP	Biopsy sampling	4	20/172	11.6
de Benito Sanz et al. (2022)[28]	5-9	Adenomas, carcinoma(s), SSLs, HPs, other	HSP	Biopsy sampling	2	23/385	6.0
Meng et al. (2022)[30]	4-9	Adenomas, SSLs	HSP	Biopsy sampling	Unspecified	10/147	6.8
Pedersen et al. (2022)[31]	4-9	Adenomas, SSLs, HPs, other	HSP	Biopsy sampling	2-3†	34/318	10.7
Rex et al. (2022)[32]	6-9	Adenomas, SSLs, HPs, other	HSP	Biopsy sampling	4	0/36	0
Papastergiou et al. (2018)[37]	6-10	Adenomas, SSLs, HPs	CS-EMR	Biopsy sampling	5	6/83	7.2
Li et al. (2020)[27]	6-10	Adenomas, carcinoma(s), SSLs	CS-EMR	Biopsy sampling	3	2/87	2.3
Rex et al. (2022)[32]	6-9	Adenomas, SSLs, HPs, other	CS-EMR	Biopsy sampling	4	0/46	0
Kim et al. (2023)[34]	6-10	Adenomas, carcinoma(s), SSLs, HPs	CS-EMR	Biopsy sampling	2	17/200	8.5
Motchum et al. (2023)[24]	6-9	Adenomas, SSLs, HPs	CS-EMR	Biopsy sampling	2	4/64	6.3
Kim et al. (2018)[36]	5-9	Adenomas, SSLs, HPs	HS-EMR	Biopsy sampling	4	13/181	7.2
Papastergiou et al. (2018)[37]	6-10	Adenomas, SSLs, HPs	HS-EMR	Biopsy sampling	5	3/81	3.7
Zhang et al. (2018)[26]	6-9	Adenomas, SSLs	HS-EMR	Biopsy sampling	5	3/203	1.5
Li et al. (2020)[27]	6-10	Adenomas, carcinoma(s), SSLs	HS-EMR	Biopsy sampling	3	0/77	0
Rex et al. (2022)[32]	6-9	Adenomas, SSLs, HPs, other	HS-EMR	Biopsy sampling	4	0/33	0

IRR, incomplete resection rate; HPs, hyperplastic polyps; BFP, biopsy forceps polypectomy; NA, not applicable; CSP, cold snare polypectomy; SSLs, sessile serrated lesions; HSP, hot snare polypectomy; CS-EMR, cold snare endoscopic mucosal resection; HS-EMR, hot snare endoscopic mucosal resection; *Inclusion of SSLs and/or assignment of SSLs to either the adenomas or HPs group unspecified; †Polyps 4-6 mm: 2 biopsies, polyps 7-9 mm: 3 biopsies.

Table 7s Incomplete resection rate as reported in the literature for polyps ~10-19 mm, grouped by polyp resection device and technique.

Author (year)	Size of included polyps, mm	Histological subtype(s) of included polyps	Resection device / technique	Method for assessment of IRR	Number of biopsies	Incompletely resected polyps, n	IRR, %
Li et al. (2020)	11-20	Adenomas, carcinoma(s), SSLs	CSP	Biopsy sampling	5	32/132	24.2
Ma et al. (2022)	10-15	Adenomas, SSLs	CSP	Biopsy sampling	4	3/87	3.4
Rex et al. (2022)	10-15	Adenomas, SSLs, HPs, other	CSP	Biopsy sampling	4	0/27	0
Mangira et al. (2023)	10-19	Adenomas, SSLs, HPs	CSP	Biopsy sampling	4-8+*	4/45	8.9
von Renteln et al. (2023)	10-20	Adenomas, carcinomas, SSLs, HPs, other	CSP	Biopsy sampling	4	3/14	21.4
Pohl et al. (2013)	10-20	Adenomas, carcinoma(s), SSLs	HSP	Biopsy sampling	4	19/110	17.3
Rex et al. (2022)	10-15	Adenomas, SSLs, HPs, other	HSP	Biopsy sampling	4	2/35	5.7
Li et al. (2020)	11-20	Adenomas, carcinoma(s), SSLs	CS-EMR	Biopsy sampling	5	13/165	7.9
Rex et al. (2022)	10-15	Adenomas, SSLs, HPs, other	CS-EMR	Biopsy sampling	4	1/35	2.9
Mangira et al. (2023)	10-19	Adenomas, SSLs, HPs	CS-EMR	Biopsy sampling	4-8+*	3/305	1.0
Motchum et al. (2023)	10-20	Adenomas, SSLs, HPs	CS-EMR	Biopsy sampling	4	1/32	3.1
Li et al. (2020)	11-20	Adenomas, carcinoma(s), SSLs	HS-EMR	Biopsy sampling	5	12/190	6.3
Rex et al. (2022)	10-15	Adenomas, SSLs, HPs, other	HS-EMR	Biopsy sampling	4	4/32	12.5

IRR, incomplete resection rate; SSLs, sessile serrated lesions; CSP, cold snare polypectomy; HPs, hyperplastic polyps; HSP, hot snare polypectomy; CS-EMR, cold snare endoscopic mucosal resection; HS-EMR, hot snare endoscopic mucosal resection; *En bloc resections: 4 biopsies, piecemeal resections: 8 biopsies and at areas of snare overlap.

Fig. 1s Study screening and selection flow chart.

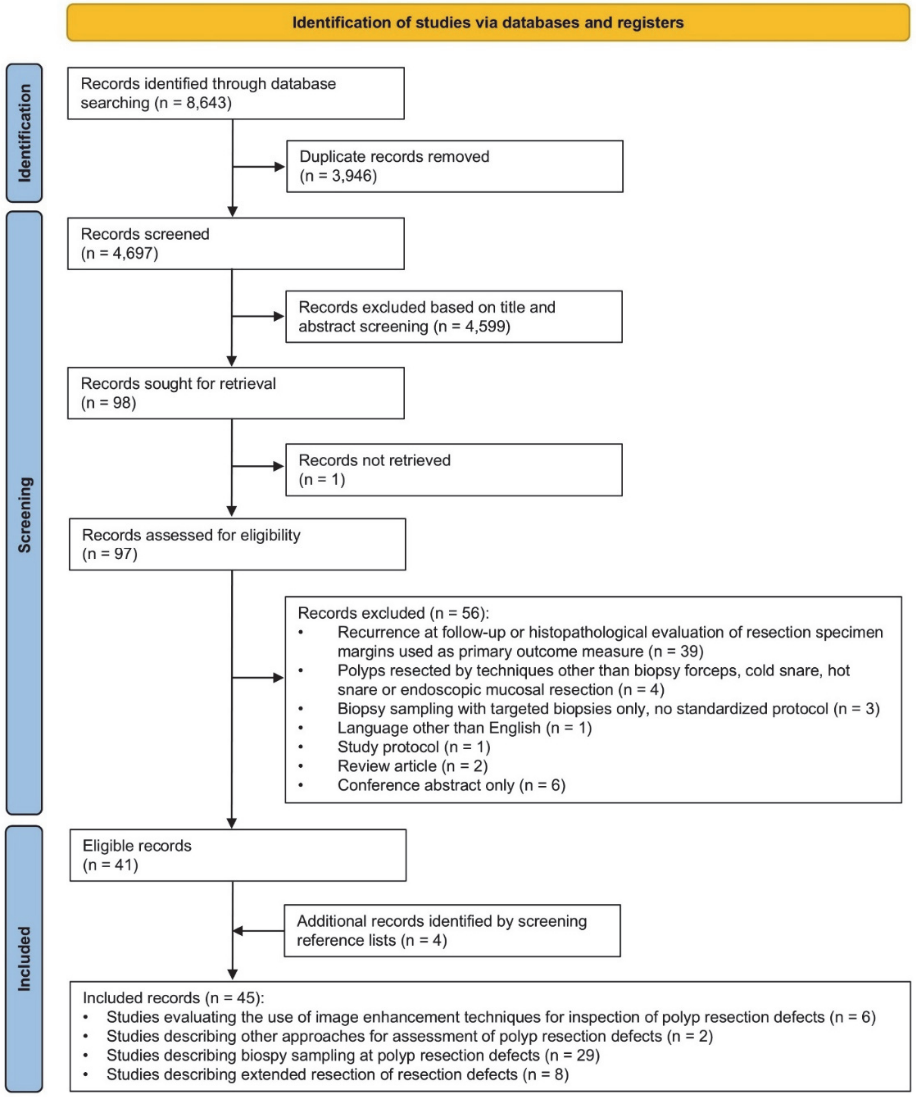

SUPPLEMENTARY REFERENCES

1. Shahid MW, Buchner AM, Coron E et al. Diagnostic accuracy of probe-based confocal laser endomicroscopy in detecting residual colorectal neoplasia after EMR: a prospective study. Gastrointest Endosc 2012; 75: 525-533. doi:10.1016/j.gie.2011.08.024
2. Desomer L, Tutticci N, Tate DJ et al. A standardized imaging protocol is accurate in detecting recurrence after EMR. Gastrointest Endosc 2017; 85: 518-526. doi:10.1016/j.gie.2016.06.031
3. Kandel P, Brand EC, Pelt J et al. Endoscopic scar assessment after colorectal endoscopic mucosal resection scars: when is biopsy necessary (EMR Scar Assessment Project for Endoscope (ESCAPE) trial). Gut 2019; 68: 1633-1641. doi:10.1136/gutjnl-2018-316574
4. Zorron Cheng Tao Pu L, Chiam KH, Yamamura T et al. Narrow-band imaging for scar (NBI-SCAR) classification: from conception to multicenter validation. Gastrointest Endosc 2020; 91: 1146-1154.e1145. doi:10.1016/j.gie.2019.08.036
5. João M, Areia M, Pinto-Pais T et al. Can white-light endoscopy or narrow-band imaging avoid biopsy of colorectal endoscopic mucosal resection scars? A multicenter randomized single-blind crossover trial. Endoscopy 2023; 55: 601-607. doi:10.1055/a-2018-1612
6. Ortiz O, Daca-Alvarez M, Rivero-Sánchez L et al. Linked-color imaging versus high definition white-light endoscopy for evaluation of post-polypectomy scars of nonpedunculated lesions: LCI-Scar study. Endoscopy 2024; 56: 283-290. doi:10.1055/a-2204-3236
7. Kliegis L, Obst W, Bruns J et al. Can a Polyp Detection and Characterization System Predict Complete Resection? Dig Dis 2021; 40: 115-118. doi:10.1159/000516974
8. Kudo T, Horiuchi A, Kyodo R et al. Mucosal defect size predicts the adequacy of resection of ≤10 mm nonpedunculated colorectal polyps using a new cold snare polypectomy technique. Eur J Gastroenterol Hepatol 2021; 33: e484-e489. doi:10.1097/meg.0000000000002156
9. Efthymiou M, Taylor AC, Desmond PV et al. Biopsy forceps is inadequate for the resection of diminutive polyps. Endoscopy 2011; 43: 312-316. doi:10.1055/s-0030-1256086
10. Draganov PV, Chang MN, Alkhasawneh A et al. Randomized, controlled trial of standard, large-capacity versus jumbo biopsy forceps for polypectomy of small, sessile, colorectal polyps. Gastrointest Endosc 2012; 75: 118-126. doi:10.1016/j.gie.2011.08.019
11. Jung YS, Park JH, Kim HJ et al. Complete biopsy resection of diminutive polyps. Endoscopy 2013; 45: 1024-1029. doi:10.1055/s-0033-1344394
12. Gómez V, Badillo RJ, Crook JE et al. Diminutive colorectal polyp resection comparing hot and cold snare and cold biopsy forceps polypectomy. Results of a pilot randomized, single-center study (with videos). Endosc Int Open 2015; 3: E76-80. doi:10.1055/s-0034-1390789
13. Kim JS, Lee BI, Choi H et al. Cold snare polypectomy versus cold forceps polypectomy for diminutive and small colorectal polyps: a randomized controlled trial. Gastrointest Endosc 2015; 81: 741-747. doi:10.1016/j.gie.2014.11.048
14. Park SK, Ko BM, Han JP et al. A prospective randomized comparative study of cold forceps polypectomy by using narrow-band imaging endoscopy versus cold snare polypectomy in patients with diminutive colorectal polyps. Gastrointest Endosc 2016; 83: 527-532.e521. doi:10.1016/j.gie.2015.08.053
15. O'Connor SA, Brooklyn TN, Dunckley PD et al. High complete resection rate for pre-lift and cold biopsy of diminutive colorectal polyps. Endosc Int Open 2018; 6: E173-e178. doi:10.1055/s-0043-121874
16. Perrod G, Perez-Cuadrado-Robles E, Coron E et al. Comparison of cold biopsy forceps vs cold snare for diminutive colorectal polyp removal: A multicenter non-inferiority randomized controlled trial. Clin Res Hepatol Gastroenterol 2022; 46: 101867. doi:10.1016/j.clinre.2022.101867
17. Wei MT, Louie CY, Chen Y et al. Randomized Controlled Trial Investigating Cold Snare and Forceps Polypectomy Among Small POLYPs in Rates of Complete Resection: The TINYPOLYP Trial. Am J Gastroenterol 2022; 117: 1305-1310. doi:10.14309/ajg.0000000000001799
18. Huh CW, Kim JS, Choi HH et al. Jumbo biopsy forceps versus cold snares for removing diminutive colorectal polyps: a prospective randomized controlled trial. Gastrointest Endosc 2019; 90: 105-111. doi:10.1016/j.gie.2019.01.016
19. Desai S, Gupta S, Copur-Dahi N et al. A prospective randomized study comparing jumbo biopsy forceps to cold snare for the resection of diminutive colorectal polyps. Surg Endosc 2020; 34: 1206-1213. doi:10.1007/s00464-019-06874-z

20. Yamasaki Y, Harada K, Yamamoto S et al. Evaluation of complete cold forceps polypectomy resection rate for 3- to 5-mm colorectal polyps. Dig Endosc 2021; 33: 948-954. doi:10.1111/den.13895
21. Matsuura N, Takeuchi Y, Yamashina T et al. Incomplete resection rate of cold snare polypectomy: a prospective single-arm observational study. Endoscopy 2017; 49: 251-257. doi:10.1055/s-0043-100215
22. Dwyer JP, Tan JYC, Urquhart P et al. A prospective comparison of cold snare polypectomy using traditional or dedicated cold snares for the resection of small sessile colorectal polyps. Endosc Int Open 2017; 5: E1062-e1068. doi:10.1055/s-0043-113564
23. von Renteln D, Djinbachian R, Benard F et al. Incomplete resection of colorectal polyps of 4-20 mm in size when using a cold snare, and its associated factors. Endoscopy 2023; 55: 929-937. doi:10.1055/a-1978-3277
24. Motchum L, Djinbachian R, Rahme E et al. Incomplete resection rates of 4- to 20-mm non-pedunculated colorectal polyps when using wide-field cold snare resection with routine submucosal injection. Endosc Int Open 2023; 11: E480-e489. doi:10.1055/a-2029-2392
25. Kawamura T, Takeuchi Y, Asai S et al. A comparison of the resection rate for cold and hot snare polypectomy for 4-9 mm colorectal polyps: a multicentre randomised controlled trial (CRESCENT study). Gut 2018; 67: 1950-1957. doi:10.1136/gutjnl-2017-314215
26. Zhang Q, Gao P, Han B et al. Polypectomy for complete endoscopic resection of small colorectal polyps. Gastrointest Endosc 2018; 87: 733-740. doi:10.1016/j.gie.2017.06.010
27. Li D, Wang W, Xie J et al. Efficacy and safety of three different endoscopic methods in treatment of 6-20 mm colorectal polyps. Scand J Gastroenterol 2020; 55: 362-370. doi:10.1080/00365521.2020.1732456
28. de Benito Sanz M, Hernández L, Garcia Martinez MI et al. Efficacy and safety of cold versus hot snare polypectomy for small (5-9 mm) colorectal polyps: a multicenter randomized controlled trial. Endoscopy 2022; 54: 35-44. doi:10.1055/a-1327-8357
29. Ma X, Feng X, Li Y et al. A Comparison of Incomplete Resection Rate of Large and Small Colorectal Polyps by Cold Snare Polypectomy. Clin Gastroenterol Hepatol 2022; 20: 1163-1170. doi:10.1016/j.cgh.2021.11.010
30. Meng QQ, Rao M, Gao PJ. Effect of cold snare polypectomy for small colorectal polyps. World J Clin Cases 2022; 10: 6446-6455. doi:10.12998/wjcc.v10.i19.6446
31. Pedersen IB, Rawa-Golebiewska A, Calderwood AH et al. Complete polyp resection with cold snare versus hot snare polypectomy for polyps of 4-9 mm: a randomized controlled trial. Endoscopy 2022; 54: 961-969. doi:10.1055/a-1734-7952
32. Rex DK, Anderson JC, Pohl H et al. Cold versus hot snare resection with or without submucosal injection of 6- to 15-mm colorectal polyps: a randomized controlled trial. Gastrointest Endosc 2022; 96: 330-338. doi:10.1016/j.gie.2022.03.006
33. Wei MT, Louie CY, Chen Y et al. Randomized controlled trial investigating use of submucosal injection of EverLift™ in rates of complete resection of non-pedunculated 4-9 mm polyps. Int J Colorectal Dis 2022; 37: 1273-1279. doi:10.1007/s00384-022-04136-4
34. Kim MJ, Na SY, Kim JS et al. Cold snare polypectomy versus cold endoscopic mucosal resection for small colorectal polyps: a multicenter randomized controlled trial. Surg Endosc 2023; 37: 3789-3795. doi:10.1007/s00464-023-09875-1
35. Pohl H, Srivastava A, Bensen SP et al. Incomplete polyp resection during colonoscopy-results of the complete adenoma resection (CARE) study. Gastroenterology 2013; 144: 74-80.e71. doi:10.1053/j.gastro.2012.09.043
36. Kim HS, Jung HY, Park HJ et al. Hot snare polypectomy with or without saline solution/epinephrine lift for the complete resection of small colorectal polyps. Gastrointest Endosc 2018; 87: 1539-1547. doi:10.1016/j.gie.2018.01.031
37. Papastergiou V, Paraskeva KD, Fragaki M et al. Cold versus hot endoscopic mucosal resection for nonpedunculated colorectal polyps sized 6-10 mm: a randomized trial. Endoscopy 2018; 50: 403-411. doi:10.1055/s-0043-118594

Thieme

APPENDICES

Appendix 1s. Preferred Reporting Items for Systematic reviews and Meta-Analyses extension for Scoping Reviews (PRISMA-ScR) Checklist

SECTION	ITEM	PRISMA-ScR CHECKLIST ITEM	REPORTED ON PAGE #
**TITLE**			
Title	1	Identify the report as a scoping review.	1
**ABSTRACT**			
Structured summary	2	Provide a structured summary that includes (as applicable): background, objectives, eligibility criteria, sources of evidence, charting methods, results, and conclusions that relate to the review questions and objectives.	Abstract
**INTRODUCTION**			
Rationale	3	Describe the rationale for the review in the context of what is already known. Explain why the review questions/objectives lend themselves to a scoping review approach.	2
Objectives	4	Provide an explicit statement of the questions and objectives being addressed with reference to their key elements (e.g., population or participants, concepts, and context) or other relevant key elements used to conceptualize the review questions and/or objectives.	2, 3, Table S1
**METHODS**			
Protocol and registration	5	Indicate whether a review protocol exists; state if and where it can be accessed (e.g., a Web address); and if available, provide registration information, including the registration number.	2-3
Eligibility criteria	6	Specify characteristics of the sources of evidence used as eligibility criteria (e.g., years considered, language, and publication status), and provide a rationale.	2-3
Information sources*	7	Describe all information sources in the search (e.g., databases with dates of coverage and contact with authors to identify additional sources), as well as the date the most recent search was executed.	2-3
Search	8	Present the full electronic search strategy for at least 1 database, including any limits used, such that it could be repeated.	Appendix S2
Selection of sources of evidence†	9	State the process for selecting sources of evidence (i.e., screening and eligibility) included in the scoping review.	2-3
Data charting process‡	10	Describe the methods of charting data from the included sources of evidence (e.g., calibrated forms or forms that have been tested by the team before their use, and whether data charting was done independently or in duplicate) and any processes for obtaining and confirming data from investigators.	2-3
Data items	11	List and define all variables for which data were sought and any assumptions and simplifications made.	4, Tables 2-4, S3-S4

SECTION	ITEM	PRISMA-ScR CHECKLIST ITEM	REPORTED ON PAGE #
Critical appraisal of individual sources of evidence§	12	If done, provide a rationale for conducting a critical appraisal of included sources of evidence; describe the methods used and how this information was used in any data synthesis (if appropriate).	N/A
Synthesis of results	13	Describe the methods of handling and summarizing the data that were charted.	2-4
**RESULTS**			
Selection of sources of evidence	14	Give numbers of sources of evidence screened, assessed for eligibility, and included in the review, with reasons for exclusions at each stage, ideally using a flow diagram.	4, Figure S1
Characteristics of sources of evidence	15	For each source of evidence, present characteristics for which data were charted and provide the citations.	4-6 and Tables 1-4, S3-S7
Critical appraisal within sources of evidence	16	If done, present data on critical appraisal of included sources of evidence (see item 12).	N/A
Results of individual sources of evidence	17	For each included source of evidence, present the relevant data that were charted that relate to the review questions and objectives.	4-6 and Tables 1-4, S3-S7
Synthesis of results	18	Summarize and/or present the charting results as they relate to the review questions and objectives.	4-6 and Tables 1-4, S3-S7
**DISCUSSION**			
Summary of evidence	19	Summarize the main results (including an overview of concepts, themes, and types of evidence available), link to the review questions and objectives, and consider the relevance to key groups.	6-10
Limitations	20	Discuss the limitations of the scoping review process.	9-10
Conclusions	21	Provide a general interpretation of the results with respect to the review questions and objectives, as well as potential implications and/or next steps.	10
**FUNDING**			
Funding	22	Describe sources of funding for the included sources of evidence, as well as sources of funding for the scoping review. Describe the role of the funders of the scoping review.	N/A

JBI = Joanna Briggs Institute; PRISMA-ScR = Preferred Reporting Items for Systematic reviews and Meta-Analyses extension for Scoping Reviews.
* Where *sources of evidence* (see second footnote) are compiled from, such as bibliographic databases, social media platforms, and Web sites.
† A more inclusive/heterogeneous term used to account for the different types of evidence or data sources (e.g., quantitative and/or qualitative research, expert opinion, and policy documents) that may be eligible in a scoping review as opposed to only studies. This is not to be confused with *information sources* (see first footnote).
‡ The frameworks by Arksey and O'Malley (6) and Levac and colleagues (7) and the JBI guidance (4, 5) refer to the process of data extraction in a scoping review as data charting.
§ The process of systematically examining research evidence to assess its validity, results, and relevance before using it to inform a decision. This term is used for items 12 and 19 instead of "risk of bias" (which is more applicable to systematic reviews of interventions) to include and acknowledge the various sources of evidence that may be used in a scoping review (e.g., quantitative and/or qualitative research, expert opinion, and policy document).
From: Tricco AC, Lillie E, Zarin W, O'Brien KK, Colquhoun H, Levac D, et al. PRISMA Extension for Scoping Reviews (PRISMAScR): Checklist and Explanation. Ann Intern Med. 2018;169:467–473. doi: 10.7326/M18-0850

.**Appendix 2s Search strategy and results**

**Database**	**Results (n)**	**After deduplication (n)**
MEDLINE (PubMed)	2,765	2,729
Embase	3,425	1,116
Web of Science	1,893	491
Cochrane	560	361
**Total**	**8,643**	**4,697**

MEDLINE/PubMed (2,765 results)

**Search**	**Query**	**Results**
#1	Search: **("Colonoscopy"[MeSH Terms] OR "Endoscopy"[MeSH Terms] OR "colonoscop*"[Title/Abstract] OR "endoscop*"[Title/Abstract]) AND ("Intestinal Polyps"[MeSH Terms] OR ("Colorectal Neoplasms"[MeSH] AND (lesion*[tiab] OR neoplasm*[tiab])) OR "polyp*"[Title/Abstract]) AND ("polypectom*"[Title/Abstract] OR "resection*"[Title/Abstract] OR "remov*"[Title/Abstract]) AND ("incomplete resection*"[Title/Abstract] OR "complete resection*" [Title/Abstract] OR "incomplete remov*"[Title/Abstract] OR "complete remov*"[Title/Abstract] OR "incomplete polypectom*"[Title/Abstract] OR "complete polypectom*"[Title/Abstract] OR "irradical resection*"[Title/Abstract] OR "radical resection*"[Title/Abstract] OR "irradical remov**"[Title/Abstract] OR "radical remov*"[Title/Abstract] OR "irradical polypectom*"[Title/Abstract] OR "radical polypectom*"[Title/Abstract] OR "completeness"[Title/Abstract] OR "radicality" [Title/Abstract] OR "recurrence*"[Title/Abstract] OR "post colonoscopy colorectal cancer*" [Title/Abstract] OR "PCCRC" [Title/Abstract]) AND ("Incidence"[MeSH] OR "Prevalence"[MeSH] OR "Diagnosis"[MeSH] OR "diagnosis" [Subheading] OR "incidence*"[tiab] OR "prevalen*" OR**	2,765

Search	Query	Results
	"rate"[tiab] OR "assess*"[Title/Abstract] OR "diagnos*"[Title/Abstract] OR "measure*"[Title/Abstract] OR "determin*"[Title/Abstract])	

Embase (3,425 results)

Search	Query	Results
#2	#1 NOT ('conference abstract'/it OR 'conference review'/it OR 'conference paper'/it OR 'editorial'/it OR 'erratum'/it OR 'letter'/it OR 'note'/it OR 'short survey'/it OR 'tombstone'/it OR 'chapter'/it)	3,425
#1	('colonoscopy'/exp OR 'endoscopy'/exp OR ("colonoscop*" OR "endoscop*"):ti,ab,kw) AND ('intestine polyp'/exp OR ('colorectal tumor'/exp AND (lesion*:ti,ab,kw OR neoplasm*:ti,ab,kw)) OR "polyp*":ti,ab,kw) AND ("polypectom*" OR "resection*" OR "remov*"):ti,ab,kw AND ("incomplete resection*" OR "complete resection*" OR "incomplete remov*" OR "complete remov*" OR "incomplete polypectom*" OR "complete polypectom*" OR "irradical resection*" OR "radical resection*" OR "irradical remov**" OR "radical remov*" OR "irradical polypectom*" OR "radical polypectom*" OR "completeness" OR "radicality" OR "recurrence*" OR "post colonoscopy colorectal cancer*" OR "PCCRC"):ti,ab,kw AND ('incidence'/exp OR 'prevalence'/exp OR 'diagnosis'/exp OR ("incidence*" OR "prevalen*" OR "rate" OR "assess*" OR "diagnos*" OR "measure*" OR "determin*"):ti,ab,kw)	

World of Science (1,893 results)

Search	Query	Results
#1	**TS=(<br>("colonoscop*" OR "endoscop*")<br>AND<br>(("Colorectal Neoplasms" AND (lesion* OR neoplasm*)) OR "polyp*")<br>AND<br>("polypectom*" OR "resection*" OR "remov*")<br>AND<br>("incomplete resection*" OR "complete resection*" OR "incomplete remov*" OR "complete remov*" OR "incomplete polypectom*" OR "complete polypectom*" OR "irradical resection*" OR "radical resection*" OR "irradical remov**" OR "radical remov*" OR "irradical polypectom*" OR "radical polypectom*" OR "completeness" OR "radicality" OR "recurrence*" OR "post colonoscopy colorectal cancer*" OR "PCCRC")<br>AND<br>("incidence*" OR "prevalen*" OR "rate" OR "assess*" OR "diagnos*" OR "measure*" OR "determin*")**	1,893

Thieme

Cochrane library (560 results)

Search	Query	Results
#1	(((colonoscop*) OR (endoscop*)) AND (((Colorectal NEXT Neoplasm*) AND (lesion* OR neoplasm*)) OR (polyp*)) AND ((polypectom*) OR (resection*) OR (remov*)) AND ((incomplete NEXT resection*) OR (complete NEXT resection*) OR (incomplete NEXT remov*) OR (complete NEXT remov*) OR (incomplete NEXT polypectom*) OR (complete NEXT polypectom*) OR (irradical NEXT resection*) OR (radical NEXT resection*) OR (irradical NEXT remov**) OR (radical NEXT remov*) OR (irradical NEXT polypectom*) OR (radical NEXT polypectom*) OR (completeness) OR (radicality) OR (recurrence*) OR (post NEXT colonoscopy NEXT colorectal NEXT cancer*) OR (PCCRC)) AND ((incidence*) OR (prevalen*) OR (rate) OR (assess*) OR (diagnos*) OR (measure*) OR (determin*)))	560
